# Adding video-debriefing to Helping-Babies-Breathe training enhanced retention of neonatal resuscitation knowledge and skills among health workers in Uganda: a cluster randomized trial

**DOI:** 10.1080/16549716.2020.1743496

**Published:** 2020-06-11

**Authors:** Beatrice Odongkara, Thorkild Tylleskär, Nicola Pejovic, Vincentina Achora, David Mukunya, Grace Ndeezi, James K. Tumwine, Victoria Nankabirwa

**Affiliations:** a Department of Paediatrics and Child Health, Gulu University Faculty of Medicine, Gulu, Uganda; b Center for International Health, University of Bergen, Bergen, Norway; c College of Health Sciences, School of Medicine, Department of Paediatrics and Child Health, Makerere University, Kampala, Uganda; d Department of Neonataology, Sachs’ Children and Youth Hospital, Stockholm, Sweden; e College of Health Sciences, School of Medicine, Department of Obstetrics and Gynaecology, Makerere University, Kampala, Uganda; f College of Health Sciences, School of Public Health, Makerere University, Kampala, Uganda

**Keywords:** Video-debriefing, Helping-Babies-Breathe, standard training, knowledge and skills, attainment and retention

## Abstract

**Background:**

Skilled birth attendants must be competent to provide prompt resuscitation to save newborn lives at birth. Both knowledge and skills (competence) decline with time after training but the optimal duration for refresher training among frontline-skilled birth attendants in low-resource settings is unknown.

**Objectives:**

We assessed the effect of an innovative Helping-Babies-Breathe simulation-based teaching method using video-debriefing compared to standard Helping-Babies-Breathe training on 1) neonatal resuscitation knowledge and skills attainment and 2) competence retention among skilled birth attendants in Northern Uganda.

**Methods:**

A total of 26 health facilities with 86 birth attendants were equally randomised to intervention and control arms. The 2nd edition of the American Association of Pediatrics Helping-Babies-Breathe curriculum was used for training and assessment. Knowledge and skills were assessed pre- and post-training, and during follow-up at 6 months. A mixed effects linear regression model for repeated measures was used to assess the short and long-term effects of the intervention on neonatal resuscitation practices while accounting for clustering.

**Results:**

Eighty-two (95.3%) skilled birth attendants completed follow-up at 6 months. Approximately 80% of these had no prior Helping-Babies-Breathe training and 75% reported practicing neonatal resuscitation routinely. Standard Helping-Babies-Breathe training with video-debriefing improved knowledge and skills attainment post-training [adjusted mean difference: 5.34; 95% CI: 0.82–10.78] and retention [adjusted mean difference: 2.97; 95% CI: 1.52–4.41] over 6 months post-training compared to standard training after adjusting for confounding and clustering. Factors that reduced knowledge and skills retention among birth attendants were monthly resuscitation of one neonate or more and being in service for more than 5 years.

**Conclusion:**

Adding video-debriefing to standard Helping-Babies-Breathe training had an effect on birth attendants’ competence attainment and retention over 6 months in Uganda. However, more research is needed to justify the proposed intervention in this context.

## Background

Despite the global effort to improve knowledge and skills among frontline-skilled birth attendants (SBAs), the reduction in neonatal mortality – especially in low-resource settings including Uganda – has been modest [1]. Uganda is committed to the global Sustainable Development Goal (SDG) 3.2 of reducing neonatal mortality to <12 per 1000 live births by 2030. To achieve this, innovation and creativity in training methods are needed. Methods such as video-debriefing can potentially enhance neonatal resuscitation knowledge and skills attainment, and retention among SBAs.

Debriefing is a process of information stimulus and response used by highly skilled professionals working in high-risk industries such as aviation, army, and healthcare systems, to improve behaviour or performance and promote clients and patients’ safety [2,3]. Video-debriefing is the use of post-event video recordings to facilitate debriefing and learning among frontline SBAs. An SBA is a formally trained health-worker who provides skilled care to pregnant mothers during delivery.

Globally, about 10% of neonates require support to establish breathing at birth. Of these, >90% can be saved with low-cost interventions, such as the Helping-Babies-Breathe (HBB) training program. The HBB program is simulation-based training that utilizes neonatal simulators known as NeoNatalie manikin (Laerdal Global, Stavanger, Norway) to impart neonatal resuscitation knowledge and skills among SBAs in low-resource settings [1]. The 2nd edition of the standard American Association of Pediatrics (AAP) HBB curriculum consists of principles of basic neonatal resuscitation, a multiple-choice questionnaire (MCQ) on knowledge, and bag-mask ventilation (BMV) and objective structured clinical examinations A and B (OSCE-A & B) skills checklists [4,5].

Since the introduction of the HBB programme in 2010, many SBAs in low-resource settings have been trained and thousands of newborn babies have received neonatal resuscitation. While many studies have documented a decline in knowledge and skills with time after HBB training, the rate of knowledge and skills decline and the optimal timing for instituting refresher training are unknown [6–8].

Furthermore, several studies demonstrate conflicting benefits of the HBB training program to the attained knowledge and skills of neonatal care practices and survival. A study in Tanzania showed no knowledge and skills translation into neonatal care practice post-training [9]. A systematic review reported improved neonatal survival within the first 24 h of life but was un-sustained at 28 days of life [10]. The relative rarity of birth asphyxia and the opportunity to practice neonatal resuscitation skills by trained SBAs may explain this paucity of knowledge and skills [11,12]. A randomized trial of a booster training strategy by hands-on or video trainings at 3–5 months among resident physicians in the United States of America (USA) showed no beneficial effects regarding the retention of knowledge and skills [13], while evidence from a longitudinal study in the Sudan showed that regular manikin practice was associated with skills retention among village midwives one year after training [14].

In view of the conflicting findings above, we hypothesized that a cluster-randomized trial of an innovative teaching method of adding video-debriefing to standard neonatal resuscitation training compared with standard training alone would improve knowledge and skills attainment and retention among SBAs in Lira district, northern Uganda, over a 6 months’ follow-up period. The main objectives of the study were to: 1) assess the effect of standard HBB training with video-debriefing compared with standard training alone on SBAs’ knowledge and skills attainment immediate post-training and 2) estimate the effect of this modified teaching method on knowledge and skills retention over 6 months’ period after training.

## Methods

We conducted a cluster-randomized trial of 26 health facility (HF) clusters (18 public and 8 private) conducting deliveries in Lira District, Northern Uganda, over a 6-month follow-up period. The district has a low proportion of health facility deliveries (<60%), and a high neonatal mortality (30/1000 live births, above the national average of 19/1000) [15]. A total of 86 SBAs from 26 HF clusters were trained in June 2018 and followed-up for 6 months from July 2018 to January 2019. A cluster design was deemed appropriate to study interventions that target a group of SBAs from the same institution with similar characteristics and behaviour while controlling for cross contamination across individuals from the same facility, had they been individually randomized.

### Sample size for clusters

To calculate the number of clusters, we assumed a fixed number of clusters, minimal intra-cluster variability, variable cluster sizes (2 to 6 SBAs each), and minimum sample size to detect a 30% difference in competence (knowledge and skills) between intervention and control arms. Adding 20% loss to follow up, a total of 26 clusters (13 in each arm), were deemed adequate [16].

*Sampling*: All trial participants providing delivery and neonatal care were selected per cluster to participate in the training using the population proportional to sample size. Most facilities, however, had between 2 and 6 SBAs. In such cases, all were included in the training program.

Restricted randomization, allocation concealment, and blinding were done by a statistician who was not part of the study. The clusters were randomized into intervention and control arms in a ratio of 1:1. The assessors/research assistants were blinded to the intervention allocations, but the study participants, the principal investigator (PI) and trainers knew the group on the day of the training. The PI and assessment team were blinded to the HF intervention allocation throughout the follow-up period, by the data manager who kept the randomization codes. This controlled both performance and assessment bias.

### Inclusion and exclusion criteria

We included HFs and SBAs providing delivery and newborn care services. Community vaccinators and laboratory technicians who turned up for training and were neither providing delivery nor newborn care were excluded.

## Description of interventions

The control arm received standard HBB training alone. The intervention arm received video-debriefing in addition to the standard HBB training.

### The control (standard HBB training) arm

International, national and regional HBB facilitators trained the SBAs using the 2nd edition of the AAP HBB training curriculum for 2 days. On Day 1 of the training, all SBAs received pre-test knowledge and skills assessments in the order of MCQs, BMV, OSCE-A and OSCE-B, respectively. The pre-test was followed by integrated lectures and demonstrations on neonatal resuscitation skills. The topics covered during the training were: 1) the current global status of newborn health and the burden of neonatal morbidity and mortality, 2) birth preparedness in the labour suit, and 3) care of the healthy, sick and very sick newborn who require resuscitation and/or referral care. Question and answer sessions followed the lectures. The SBAs were then divided into three groups of 6–8 for further practical demonstrations and group practice of birth preparedness, ventilation skills, care of both healthy and sick newborn. A total of 6 h (3 h each day) was allowed for skills practice. Each group spent 2 h in each of the three skills sessions. During the different practical sessions, time was given for group practice in threes (a birth attendant, a mother and an assistant). The participants could ask the trainers and PI questions and clarifications on some difficult practical skills techniques. On the second day of the training, after all the SBAs were satisfied with the acquired resuscitation skills techniques, a post-test assessment was given in a similar way as the pre-test. Ongoing training was assessed at the end of each day using the Kirkpatrick training assessment tool to improve the quality of training and maximize learning [17].

### Intervention arm (standard HBB training and video-debriefing)

In addition to the standard HBB training, the intervention arm had their HBB simulation sessions video-recorded and used for debriefing. Participants were divided into two groups. One group remained in the video-debriefing session, while the other went for practical skills sessions as described in the standard HBB training alone. During debriefing, participants also worked in teams of threes (a birth attendant, a mother and an assistant). Prior to the debriefing, the participants were asked to set learning objectives at the beginning of each practical session using the SHARP (Set learning objectives, How it went, Address concerns, Review learning points, Plan ahead) debriefing tool [18]. At the end of each practice session, SBAs were asked how the session had gone and concerns arising from the practice were addressed. In addition, the learning objectives were reviewed, and the participants planned for improved performance. This was followed by viewing of the video recording by the group, with learning points and feedback being given by the participants in the simulation scenario, followed by the rest of the group members and the facilitator. After watching the video, the next team had their practice sessions. During each session, the facilitator read the case scenarios aloud. The team simulated this while being videotaped. This was done until every participant had had his/her turn to be a birth attendant. The objective assessment of debriefing (OSAD) tool was used to guide the facilitators during debriefing sessions [18].

Debriefing was done in a separate room from the HBB skills training rooms with participants and two debriefing leaders/facilitators in attendance. As in the control arm, all the participants in the intervention group were encouraged to practice while asking the facilitators questions and seeking clarification. Finally, post-training knowledge and skills assessment were given to the SBAs in the same way as in the control arm.

### Knowledge and skills assessment

Knowledge and skills attainment were defined as the percentage scores in knowledge and skills tests in the immediate post-training period. Skills assessments were done using validated HBB program tools (BMV, OSCE-A and OSCE-B checklists] for assessing neonatal resuscitation skills among SBAs using NeoNatalie manikin [5]. Knowledge was assessed using the standardised HBB MCQs. Assessments were done pre- and post-intervention, and during subsequent longitudinal follow-up at 1, 3 and 6 months. The skills scores were obtained by taking the means scores for BMV, OSCE-A and OSCE-B. Scores were presented in percentages and analysed as continuous variables.

### Outcome variables

The two outcomes measured were 1) knowledge and skills attainment in the immediate post-training period, and 2) knowledge and skills retention over a 6-month follow-up period.

### Independent variables (covariates)

Data were collected on the socio-demographic characteristics of SBAs (age, sex, educational qualifications and occupation), health unit type, number of deliveries at the health unit, HBB training experience, number of HBB training sessions attended and duration since last training, number of years spent in services, monthly number of neonatal resuscitations conducted prior to training, routine newborn resuscitation practices, and routine delivery care in the past 6 months. The occupation of the health workers was categorised as nurses/midwives, and clinical officers/doctors. Qualification was defined as the highest attained level of education: certificate, diploma, bachelor’s degree, master’s degree, and categorised as certificate, diploma or degree. HBB training experience was recorded as ‘yes’ if the person had ever attended at least one training. The duration since last training was recorded in months. Routine delivery and resuscitation practices were recoded as ‘yes’ if one provided delivery and neonatal resuscitation care at one’s facilities on a regular basis or daily. The number of resuscitations per facility was counted from the birth registers and recorded as the number of babies resuscitated which was subsequently categorized as none, one or more. Each health worker was also asked to record the number of babies he/she had resuscitated in the previous month prior to the training. The number of deliveries was physically counted as the total number delivered per facility and health workers were also asked to record the average monthly number of deliveries attended and these were categorized as none, 1 to 9 and 10 or more.

### Quality control

Research assistants were trained, and the instruments pre-tested. The HBB trainers were nationally trained facilitators. The PI and research assistants were trained in neonatal resuscitation, assessment methods and debriefing by a master trainer from Sachs’ Children and Youth Hospital, Stockholm, Sweden. Both internal and external validity, and reliability of the OSCE scores, were checked by the PI who participated in a few of the skills sessions while making independent observations.

### Data management and analysis

The Data were collected using standardized HBB knowledge (MCQ) and skills (BMV and OSCE-A & B) assessment tools. The data were entered using EPI Data 3.1 (EpiData Association; Enghavevej 34, DK5230 Odense M, Denmark) and exported to STATA Version 14 (StataCorp; College Station, TX, USA) for analysis.

Intention to treat analysis was done. At bivariable analysis, baseline categorical variables were summarized into proportions and presented in a table. Chi-squared tests were in bivariable analysis to screen for significant differences in baseline SBAs’ sociodemographic and HF characteristics between intervention and control arms. Continuous variables were summarized as means with standard error. The mean differences between the two arms (intervention and control) were compared using two sample t-tests and the results presented in a table. The years in service and monthly number of resuscitations conducted which had *P*-value <0.10 at baseline bivariable analysis were included in the multilevel mixed effects linear regression model, in order to control for differences in baseline characteristics, clustering and repeated measurements from the same SBAs over time. Stratified analysis and adjustment in multivariable analysis for confounding were carried out. A factor was deemed confounding if 1) the crude and adjusted mean difference in scores deferred by ≥10%, and/or 2) the crude mean difference was outside the strata-specific mean difference ranges or known apriori (sex, age, and prior HBB training). The fixed and random effects were intervention and health facility clusters, respectively. The statistical significance level was set at a *P-value* < 0.05.

### Ethics

Ethical clearance was obtained from the Makerere University School of Medicine Research and Ethics Committee (SOMREC), reference number 2015–085, and the Uganda National Council of Science and Technology (UNCST), reference number HS 2478, the Ministry of Health through Lira District Health Office and health facility administrations. Clearance was also sought from the Norwegian Research Council. Assessment was done by the Norwegian Regional Committee for Medical and Health Research Ethics (REK Vest). The study was found to be outside their jurisdiction and hence qualified for exemption (2018/58/REK Vest). The study was registered at ClinicalTrials.gov (NCT03703622). Written informed consent was obtained from all the trial SBAs. Informed consent was also obtained from the participants before the video-recording. SBAs were not at risk, since we used simulation-based clinical case scenarios. For fairness of participation, we included SBAs from both public and private delivery facilities and from all HFs providing delivery and newborn care. Training frontline service providers (SBAs) ensured the provision of quality delivery and newborn care to reduce neonatal mortality in the region. This paper was prepared in accordance with CONSORT guidelines [19,20].

## Results

### Trial profile

**Figure 1.**
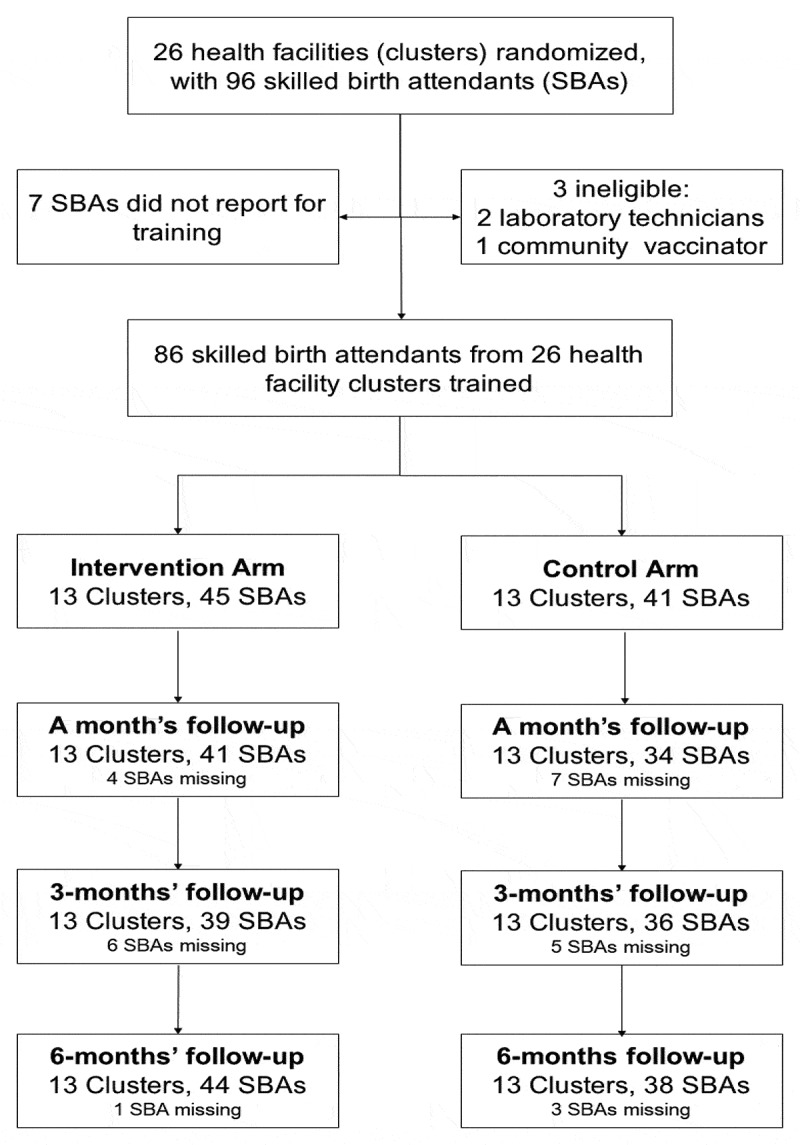
CONSORT flow chart trial participants.

The trial profile is presented in the CONSORT flow chart (Figure 1). A total of 26 HFs (clusters) were randomised into intervention or video-debriefing plus standard HBB training or control (standard HBB training only) in a ratio of 1:1 (Figure 1). Ninety-six SBAs were identified for training. After excluding seven who did not report for training and three who were providing neither delivery nor newborn care, 86 remained in the final sample. All the 26 clusters had SBAs trained and followed up for 6 months. The control arm witnessed a higher loss to follow-up throughout the study period. Follow-up at 6 months was about 95% (82/86).

### Characteristics of trial participants

The baseline characteristics were similar between groups except for the SBAs’ years spent in services (*P* = 0.04) and the monthly number of resuscitations conducted. Most of the SBAs (80%) had no prior HBB training before our intervention. Approximately 69% of the participants were from public (government) HFs and the majority of SBAs (84%) were from lower HFs (HCIIs and IIIs). Details are given in Table 1.

**Table 1.** Sociodemographic characteristics of the trial participants.

Characteristics	All n (%) N = 86	Intervention n (%) N = 45	Control n (%) N = 41	*P* value
**Sex**				
Male	13(15.1)	9(20.0)	4(9.8)	0.22
Female	73(84.9)	36(80.0)	37(90.2)	
**Qualification**				
Degree/Diploma	32(37.2)	17(77.8)	15(36.6)	0.91
Certificate	54(62.8)	28(62.2)	26(63.4)	
**Profession**				
Midwife/Nurse	77(89.5)	42(93.3)	35(85.4)	0.17
Doctor/Clinical Officer	9(10.47)	3(6.7)	6(14.6)	
**No. of Years in service**				
≤5	43(52.4)	18(40)	25(61.0)	
6–15	26(30.2)	15(33.3)	11(26.8)	0.27
15	17(19.8)	12(26.7)	5(12.2)	0.04*
**Prior HBB trained**				
Yes	17(19.8)	9(20.0)	8(19.5)	0.96
No	69(80.2)	36(80.0)	33(80.5)	
**Duration since last training**				
≤12 months	9(52.9)	4(44.4)	5(62.5)	
>12 months	8(47.1)	5(55.6)	3(37.5)	0.37
Not trained	69(80.2)	36(80.0)	33(80.5)	0.62
**Number of HBB trainings**				
once	12(14.0)	7(15.6)	5(12.2)	
2 or more	5(5.8)	2(44.4)	3(7.3)	0.54
None	69(80.2)	36(80.0)	33(80.5)	0.74
**Health Facility type**				
Public	59(68.6)	39(86.7)	20(48.8)	0.12
Private	27(31.4)	6(13.3)	21(51.2)	
**Health Facility level**				
Health Centre IV–V	14(16.3)	9(20.0	5(12.2)	0.66
Health Centre II–III	72(83.7)	36(80.0)	36(87.8)	
**Routinely conducts delivery**				
Yes	71(82.3)	38(84.4)	33(80.5)	0.66
No	15(17.4)	7(15.6)	8(19.5)	
**Monthly no. of deliveries**				
≥10	15(20.0)	6(15.4)	9(25.0)	
<10	60(69.8)	33(73.3)	37(65.9)	0.39
None	11(12.8)	6(23.3)	5(12.2)	0.49
**Routinely resuscitates babies**				
Yes	63(75.0)	36(80.0)	27(69.2)	0.27
No	21(25.0)	9(20.0)	12(30.8)	
**Monthly no. of resuscitations**				
>1	19(22.1)	7(15.6)	12(29.3)	
1	55(64.0)	33(73.3)	22(53.7)	0.09
None	12(14.0	5(11.1)	7(17.1)	0.81

*p < 0.05 indicates significant baseline difference between intervention and control arms.

## Effects of video-debriefing on skills attainment and retention up to 6-months post-training

### Knowledge and skills attainment

Adding video-debriefing to standard HBB training had a significant effect on skills and the combined knowledge and skills (competence) attainment in the immediate post-training period after adjusting for baseline characteristics. Details are summarized in Table 2.

**Table 2.** Bivariable and multivariable analysis for effect of video-debriefing on knowledge and skills scores at different time points.

	Intervention Mean (SE)	Control Mean (SE)	(Intervention – Control) Mean diff. (95% CI)	Adjusted*^a^* Mean diff (95% CI)	*P* value
Knowledge					
Pretest	81.35(1.98)	78.04(2.19)	3.31(−2.54–9.16)	1	
Post test	91.35(1.43)	89.16(2.66)	2.20(−3.60–7.99)	3.96(−1.60–9.52)	0.162
1 month	87.88(1.54)	86.71(1.56)	1.17(−3.23–5.57)	2.33(−2.07–6.73)	0.300
3 months	91.48(1.061)	91.93(1.16)	0.45(−3.60–2.69)	0.65(−2.36–3.66)	0.673
6 months	91.25(1.47)	90.19(1.56)	1.06(−3.23–5.34)	2.02(−2.02–6.01)	0.326
Bag Mask Ventilation					
Pretest	39.05(3.38)	40.50(4.09)	−1.45(−11.94–9.04)	1	
Post test	94.99(1.10)	85.88(3.55)	9.12(2.13–16.10)*	10.50(0.65–17.35)	0.003*
1 month	95.12(1.57)	92.69(1.96)	2.42(−2.5 4–7.36)	1.72(−3.27–6.72)	0.499
3 months	96.00(0.94)	94.12(1.29)	1.87(−1.27–5.02)	2.17(−1.17–5.53)	0.203
6 months	95.25(1.07)	91.36(2.03)	3.89(−0.54–8.32)	4.04(−1.08–9.16)	0.122
OSCE-A					
Pretest	56.33(2.98)	48.81(3.06)	7.53(−0.99–16.04)	1	
Post test	83.26(1.86)	82.05(2.81)	1.21(−5.33–7.74)	2.61(−3.67–8.88)	0.416
1 month	83.40(1.89)	83.06(2.41)	0.34(−5.69–6.37)	1.85(−3.85–7.56)	0.524
3 months	93.03(1.42)	90.49(1.92)	2.53(−2.16–7.23)	2.66(−1.96–7.27)	0.259
6 months	92.59(1.56)	89.48(1.68)	3.11(−7.67–1.45)	4.33(−0.12–8.78)	0.057
OSCE-B					
Pretest	37.45(2.15)	41.58(2.54)	−4.13(−10.72–2.46)	1	
Post test	95.50(0.77)	92.19(2.52)	3.31(−1.64–8.26)	4.30(−0.58–9.17)	0.084
1 month	90.21(0.73)	89.66(1.45)	0.55(−2.53–3.63)	0.31(−2.66–3.27)	0.838
3 months	92.68(1.19)	90.20(0.98)	2.48(−0.65–5.60)	3.56(−0.01–7.14)	0.051
6 months	92.99(1.01)	90.58(1.12)	2.41(−0.58–5.41)	2.76(−0.41–5.94)	0.087
Skills					
Pretest	44.28(2.16)	42.77(2.71)	1.50(−5.32–8.32)	1	
Post test	91.25(0.90)	86.70(2.59)	4.55(−0.61–9.70)	5.80(0.82–10.78)	0.023*
1 month	89.57(1.05)	88.47(1.55)	1.10(−2.52–4.72)	1.09(−2.47–4.65)	0.549
3 months	93.85(0.90)	91.60(1.09)	2.25(−0.55–5.04)	2.75(−0.49–6.00)	0.097
6 months	93.65(1.00)	90.52(1.40)	3.13(−0.25–6.50)	3.75(0.19–7.31)	0.039*
Knowledge & skills					
Pretest	53.55(1.89)	52.23(2.30)	1.32(−4.61–7.24)	1	
Post test	91.28(0.92)	87.32(2.56)	3.96(−1.51–9.42)	5.34(0.40–10.28)	0.034*
1 month	89.15(1.01)	88.03(1.25)	1.12(−2.09–4.33)	1.39(−1.72–4.50)	0.381
3 months	93.09(0.76)	91.69(0.93)	1.41(−0.99–3.81)	1.97(−0.65–4.59)	0.140
6 months	93.07(0.98)	90.45(1.26)	2.62(−0.55–5.80)	3.34(0.14–6.54)	0.041*

*p < 0.05, SE: Standard Error, diff.: difference. OSCE: Objective structured clinical examinations A & B, ^a^Adjusted for years in service, number of resuscitations and clustering. Intervention only explains the BMV post-test result.

### Knowledge and skills retention

Adding video-debriefing to standard HBB training had significant effects on both skills and competence (knowledge and skills) retention over the 6-month period after controlling for differences in baseline characteristics (confounding) and clustering. SBAs who resuscitated at least one baby per month and those who had more than 5 years in service had less retention of neonatal resuscitation competence during the 6-month follow-up period. The summaries of mean differences and respective 95% CI of mean differences are presented in Table 3.

**Table 3.** Bivariable and multivariable mixed effects linear model for knowledge and skills retention by intervention over 6 months.

	Intervention (Video-debriefing) Mean (SE)	Control Mean (SE)	Crude (Intervention – Control) Mean diff. (95% CI)	Adjusted*^a^* Mean diff. (95% CI)	*P* value
Knowledge	88.67(0.73)	87.01(0.94)	1.65(−0.69–3.99)	2.67(1.44–3.90)	<0.001*
Bag Mask Ventilation	83.59(1.77)	80.02(1.99)	3.58(−1.66–8.82)	3.70(−0.27–7.66)	0.068
OSCE-A	81.37(1.30)	78.10(1.59)	3.27(−0.77–7.32)	4.05(2.02–6.07)	<0.001*
OSCE-B	81.35(1.64)	79.98(1.71)	1.37(−3.30–6.03)	1.42(−1.54–4.37)	0.347
Skills	82.18(1.45)	79.46(1.66)	2.72(−1.62–7.04)	3.17(1.45–4.89)	<0.001*
Knowledge & skills	83.93(1.17)	81.36(1.38)	2.57(−0.98–6.13)	2.97(1.52–4.41)	<0.001*

*P-Value < 0.05. SE: Standard Error. mean diff.: mean difference. OSCE: Objective structured clinical examination A & B, **^a^**Adjusted for years in service, routine resuscitation practices, clustering and assessment time interval.

When we adjusted for SBAs’ age, sex, monthly number of resuscitations, prior HBB training experience, and clustering instead of years in service at 6 months, the intervention effect on knowledge and skills mean difference remained statistically significant (adjusted mean difference: 3.76; 95% CI: 0.81–6.70). Details of analyses for confounding in Appendix.

## Trends in knowledge and skills mean scores between intervention arms over time

**Figure 2.**
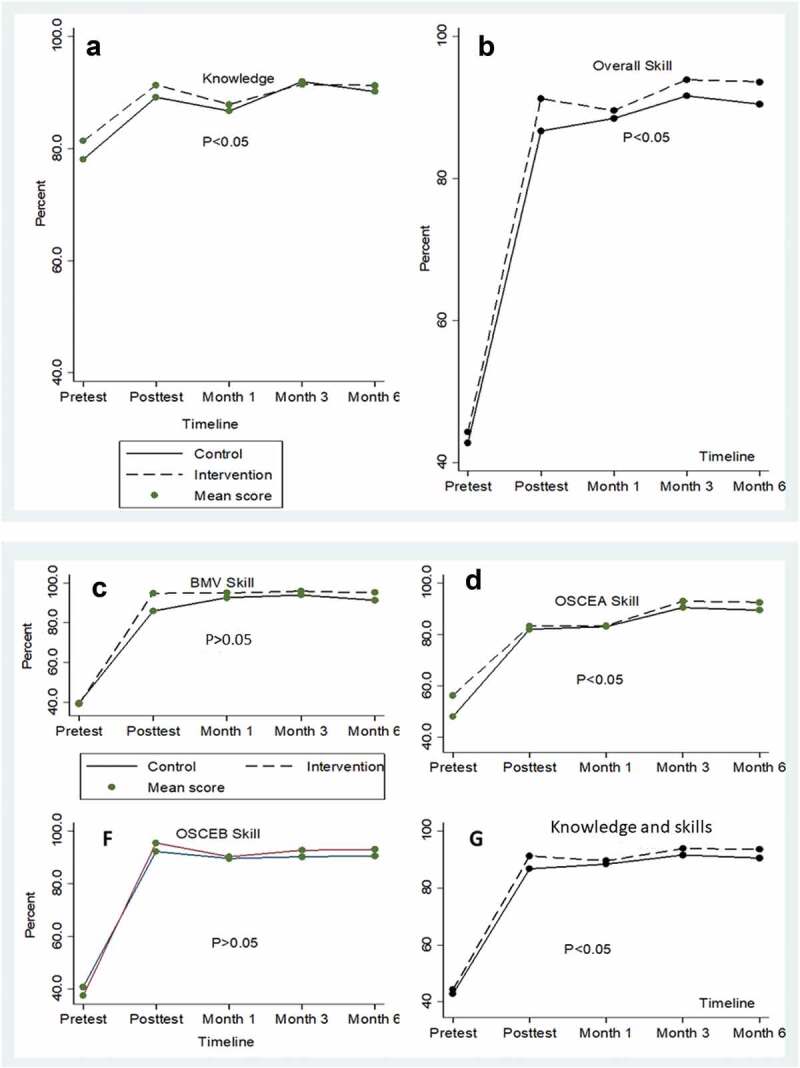
Knowledge and skills mean scores trends over 6 months.

The overall knowledge and skills mean scores in both intervention and control arms improved in the immediate post-training period. In the follow-up period, the video-debriefing arm scored higher marks throughout. There was a marked difference in knowledge and skills scores with means scores for knowledge being significantly higher than the overall and individual skills components. It is important to note that, at baseline, all SBAs scored higher in knowledge than skills. The summaries of trends are presented in Figure 2; the P-value < 0.05 showed significant differences of means scores between intervention and control arms throughout the assessment.

When analysis was done at different time points as in Table 2, significant findings following adjustment for the differences in baseline characteristics, showed higher scores in intervention groups for bag and mask ventilation in the immediate post-test period. Similar observation was also seen for skills and the overall competence at the immediate post-test period and at 6 months. In Table 3, the overall mean scores were higher among the intervention group than those in control group over the 6-month period. What this means is that when scores are compared at different time points, the intervention effect is minimal on both competence and knowledge scores. However, pooling the scores over 6 months, a statistically significant difference exists between intervention and control arms with knowledge, skills and competence being higher in the video-debriefing arm than in the control arm. An analysis of variance (ANOVA) and a generalized estimation equation (GEE) models for the pooled analysis also yielded comparable results.

## Discussion

Our study showed that SBAs in the intervention arm were more likely to attain and retain neonatal resuscitation knowledge and skills than those in the control arm in the immediate post-training period and over a 6-month period. SBAs who routinely resuscitated at least one or more neonates per month and those who had spent more than 5 years in service exhibited reduced neonatal resuscitation competence retention during the follow-up period compared to their counterparts.

Several studies worldwide have shown that neonatal resuscitation knowledge and skills decline with time post-training, with skills showing an even faster rate of deterioration than what happens to knowledge [7,9,11,12]. Therefore, HBB training alone does not guarantee skills retention several months post-training. Our findings are in agreement with numerous other studies that have shown that low-cost interventions, such as daily manikin practice, regular review meetings and clinical case reviews improve health workers’ performance, including retention of neonatal resuscitation skills [14,21,22]. The similarity of these studies with our findings could be due to repeated assessments at regular intervals which simulate quality improvement cycles reported by other studies. However, most of these studies had methodological limitations in assessing skills retention at individual levels without assessing the effect of clustering across health facilities. For example, a multicentre study in hospitals in Kenya and Nepal, reported that a combination of quality improvement cycle interventions improved neonatal resuscitation skills retention among SBAs [21]. The study relied on self-evaluation checklists filled-in by individual SBAs after every delivery and it is not clear if there were discrepancies between what was reported and what was done by the SBAs. Furthermore, the presence of surveillance officers during quality improvement cycle meetings might also have affected the SBAs behaviour, which in turn could have introduced the Hawthorne effect (observer bias) in the reported results [21].

On the other hand, the skills retention seen in our study could have been influenced by frequent assessments at close intervals that could have pressured the health workers into revising prior to each assessment, as they were given both wall-charts and participant manuals for use in their respective facilities. A study in Honduras showed that frequent OSCE skills practice among both clinic- and hospital-based staff improved skills retention after 6-month post-training [22,23]. In the same study, it was also observed that skills declined sharply at 1-month post-training. Similarly, we found a slight decline in the overall knowledge and skills scores at 1-month post-training, with the intervention arm maintaining higher scores than the control arm throughout the follow-up period. There seemed to be a dose-response effect on the measures with each assessment period. Our study findings may also add to the list of intervention combinations to improve learning and skills retention among frontline maternal newborn healthcare workers over time. Consequently, this may improve neonatal outcomes as we aim for the 2030 SDG 3 regarding the reduction of neonatal mortality to <12 per 1,000 live births by that year.

Senior SBAs with more than 5 years in service demonstrated inferior knowledge and skills retention. A possible explanation could be that the older or senior SBAs felt that they had the experience and hence were slow at taking up new changes in newborn care practices. A study by Bang Akash and colleagues (2016) reported low skills retention among senior physicians who reported being *‘too busy to practice neonatal resuscitation skills despite the provision of equipment in their facilities for daily practice’* [23]. This may, to some extent, explain our findings. We, however, did not conduct a qualitative study to ascertain the reasons for the low knowledge retention among senior SBAs in our study.

Lastly, SBAs who conducted routine neonatal resuscitation also demonstrated less knowledge and skills retention at 6 months. This finding contradicts a multicentre study from Nepal and Kenya which demonstrated a dose-response effect of refresher training and regular manikin practice on knowledge and skills retention [23]. This might be due to a perceived large workload and lack of time to read and refresh neonatal resuscitation knowledge.

### Limitations

The effect of frequent examinations of health workers could have led to improved performance and retention of neonatal resuscitation skills during the follow-up period. However, if this were the case, there would be no difference in retention between the arms. Despite the latter observation, the difference between arms remains significant. The strength of our study lies in it being a cluster-randomized trial with blinding of the assessors.

In order to minimize bias, there was explicit case definition of outcome measurements (knowledge and skills scores). Furthermore, correct addresses and telephone contacts for each participant were obtained to ensure minimal loss to follow-up. Data-cleaning was done to prevent misclassification. The calculated sample-size for individual randomization was 106, but we achieved only 86 participants in this study. This was overcome by cluster-randomization at the facility level, and all the calculated sample size of 26 clusters was followed-up for 6 months. We adjusted for differences in baseline characteristics, and clustering in the final analysis and there was very little intra-cluster variation. Studies on the validity of OSCE tool for assessment of resuscitation skills have reported fair to moderate agreement and this could have affected our scores between arms [5]. We overcame this by training our research assistants in scoring the SBAs. The interrater reliability was moderate to substantial with a kappa of 0.604 for overall skills scores.

## Conclusion

We have demonstrated that adding video-debriefing to HBB training had an effect on the overall skills and competence (combined knowledge and skills) attainment in the immediate post-training period and retention over a period of 6 months in an analysis carried out in Northern Uganda. The factors that reduced competence attainment and retention were a monthly number of resuscitations of one or more babies and years spent in service (notably more than 5 years).

### Recommendation

Debriefing is a cornerstone for simulation-based learning. If adding video-debriefing to the current standard HBB training curricula is to be justified in our context, more research is needed. A mixed method study on a bigger population should be embarked upon to assess the effectiveness of adding video-debriefing to standard HBB neonatal resuscitation training on the competence of frontline SBAs. This research should also incorporate qualitative and cost-benefit analyses. This will justify the scale up of video-debriefing for HBB in this context.

## Data Availability

Data will be available from the PI on reasonable request.

## Paper context

Neonatal mortality reduction has been modest despite mass HBB training in low-resource settings. Prior to this study, it was unknown if video-debriefing would enhance SBAs’ HBB competence in low-resource settings. However, this study revealed that adding video-debriefing to standard HBB training had some effect on competence retention over 6-month post-training compared to the usual practice. We recommend more research incorporating qualitative and cost-benefit analyses to prove its effectiveness and scalability.

## Appendix

**Table A1.** Sub group analysis of confounding factors for intervention in the immediate Post-training Bag Mask Ventilation and six-month knowledge and skills scores.

Factors (SBA)		Sub group BMV mean difference immediate at 6 months					Intervention adjusted for @ factor			Confounder or Effect Modifier
	Treatment arms mean difference		Mean difference between each factor level (Yes-No or 1–0)							
	Intervention	Control	Crude	P value	Adjusted	P value	Crude	Adjusted	% change	
Sex	3.75(1.95–5.54)	3.15(−11.43–17.73)	5.08(−0.34–10.50)	0.066	3.54(−1.24–8.31)	0.147	9.18	8.86	−3.5	
Age	0.030(−0.19–0.25)	−0.58(−1.43–0.27)	−0.16(−0.54–0.21)	0.398	−0.27(−0.70–0.16)	0.212	9.18	9.99	8.8	
Qualif2	−0.92(−5.27–3.42)	−5.27(−19.58–9.04)	−2.75(−10.07–4.56)	0.461	−3.06(−10.33–4.21)	0.409	9.18	9.11	−0.8	
Profess2	−3.21(−7.07–0.65)	−5.06(−17.15–7.02)	−3.14(−10.87–4.60)	0.427	−4.23(−11.86–3.41)	0.278	9.18	9.59	4.5	
Years in service ≤5 years	−0.41(−4.90–4.09)	13.87(−0.13–27.87)	3.81(−3.09–10.72)	0.279	6.43(−0.95–13.81)	0.088	9.18	10.50	14.4	Yes
HBB trained	2.17(−2.16–6.50)	7.27(−5.94–20.48)	4.34(−2.30–10.99)	0.200	4.54(−2.05–11.13)	0.177	9.18	9.22	0.4	Yes
Resuscitates routinely (Yes)	0.22(−2.54–2.97)	−4.65(−15.01–5.71)	−1.44(−6.35–3.47)	0.567	−1.89(−7.50–3.72)	0.509	9.18	9.44	2.8	
Routine Delivery (Yes)	1.46(−0.75–3.68)	−6.64(−17.05–3.77)	−2.26(−7.47–2.95)	0.395	−1.92(−7.23–3.39)	0.478	9.18	9.20	0.2	
# resuscitation (≥1 monthly)	0.82(−2.03–3.68)	−4.65(−15.01–5.71)	−1.02(−6.08–4.03)	0.691	−1.66(−7.52–4.20)	0.579	9.18	9.45	2.9	
	***Sub group competence mean difference immediate at 6 months***									
***Factors***	***Treatment arms mean difference***		***Mean difference between each factor level (1–0)***				***Intervention adjusted for @ factor***			*Confounding or Effect Modification*
	***Intervention***	***Control***	***Crude***	***P value***	***Adjusted***	***P value***	***Crude***	***Adjusted***	***% change***	
Sex (Male)	−5.21(−13.16–2.75)	4.38(1.14–7.62)	−1.41(−7.18–4.36)	0.632	−1.86(−7.99–4.28)	0.553	2.48	2.68	8.1	
Age in years	−0.01(−0.20–0.17)	−0.17(−0.49–0.14)	−0.06(−0.23–0.11)	0.483	−0.09(−0.71–6.28)	0.343	2.48	2.79	12.5	Yes
Qualification (Degree/Dipl)	−0.04(−5.80–5.72)	−1.15(−6.68–4.39)	−0.38(−4.35–3.59)	0.850	−0.60((−4.64–3.44))	0.770	2.48	2.51	1.2	
Profession (MW/Nurse) Yes	4.69(−12.63–22.02)	−6.14(−12.10,-0.19)	−1.88(−8.61–4.85)	0.580	−2.03(−9.35–5.30)	0.588	2.48	2.60	4.8	
Years in service ≤5 years	2.53(−1.97–7.02)	2.01(−3.56–7.58)	1.79(−1.72–5.30)	0.318	2.35(−1.16–5.86)	0.189	2.48	3.00	21.0	Yes
Prior HBB trained (Yes)	2.70(−0.86–6.26)	3.99(0.32–7.66)	2.97(0.45–5.49)	0.021	3.15(0.44–5.87)	0.023	2.48	2.56	3.2	
Resuscitates routinely (Yes)	−4.56(−7.56,-1.55)	−1.99(−6.10–2.12)	−3.12(−6.06,-0.18)	0.037	−3.17(−5.91,-0.42	0.024	2.48	2.73	25.0	Yes
Routine Delivery (Yes)	−4.73(07.55,-1.92)	−7.50(−13.53,-1.46)	−5.74(−8.73,-2.75)	<0.001	−5.70(−8.62,-2.77)	<0.001	2.48	2.53	5.0	
# resuscitation (≥1 monthly)	−4.82(−7.85,-1.79)	−1.99(−6.10–2.12)	−3.14(−6.23,-0.06)	0.046	−3.22(−6.07,-0.37)	0.027	2.48	2.80	32	Yes
# Deliveries ≥10 monthly	0.40(−3.98–4.79)	−0.33(−3.69–3.04)	−0.04(−2.87–2.79)	0.977	0.03(−2.58–2.65)	0.980	2.48	2.48	0.0	

Factors with more ≥10% difference between crude and adjusted mean difference between intervention arms was adjusted for in the multivariable mixed effects linear regression model in the immediate post-training period and at six months for bag mask ventilation competence respectively.
